# Cooperative Unfolding of Residual Structure in Heat Denatured Proteins by Urea and Guanidinium Chloride

**DOI:** 10.1371/journal.pone.0128740

**Published:** 2015-06-05

**Authors:** Ritu Singh, Md. Imtaiyaz Hassan, Asimul Islam, Faizan Ahmad

**Affiliations:** Centre for Interdisciplinary Research in Basic Sciences, Jamia Millia Islamia, New Delhi, India; Aligarh Muslim University, INDIA

## Abstract

The denatured states of proteins have always attracted our attention due to the fact that the denatured state is the only experimentally achievable state of a protein, which can be taken as initial reference state for considering the *in vitro* folding and defining the native protein stability. It is known that heat and guanidinium chloride (GdmCl) give structurally different states of RNase-A, lysozyme, α-chymotrypsinogen A and α-lactalbumin. On the contrary, differential scanning calorimetric (DSC) and isothermal titration calorimetric measurements, reported in the literature, led to the conclusion that heat denatured and GdmCl denatured states are thermodynamically and structurally identical. In order to resolve this controversy, we have measured changes in the far-UV CD (circular dichroism) of these heat-denatured proteins on the addition of different concentrations of GdmCl. The observed sigmoidal curve of each protein was analyzed for Gibbs free energy change in the absence of the denaturant (Δ*G* ^0^_X→D_) associated with the process heat denatured (X) state ↔ GdmCl denatured (D) state. To confirm that this thermodynamic property represents the property of the protein alone and is not a manifestation of salvation effect, we measured urea-induced denaturation curves of these heat denatured proteins under the same experimental condition in which GdmCl-induced denaturation was carried out. In this paper we report that (a) heat denatured proteins contain secondary structure, and GdmCl (or urea) induces a cooperative transition between X and D states, (b) for each protein at a given pH and temperature, thermodynamic cycle connects quantities, Δ*G* ^0^_N→X_ (native (N) state ↔ X state), Δ*G* ^0^_X→D_ and Δ*G* ^0^_N→D_ (N state ↔ D state), and (c) there is not a good enthalpy difference between X and D states, which is the reason for the absence of endothermic peak in DSC scan for the transition, X state ↔ D state.

## Introduction

The denatured states of proteins have always attracted our attention. This interest arises from the realization that the denatured state is the only experimentally achievable state of a protein, that can be taken as initial reference state for considering the *in vitro* folding and defining the native protein stability. However, a denatured state can play this role only if it is completely unfolded, for the presence of residual structures may influence the rate and mechanism of protein folding. This is one of the reasons why most of the efforts have gone to characterizing the guanidinium chloride (GdmCl) and urea denatured states. Classical experiments (viscosity, sedimentation, optical rotation, light scattering and hydrogen ion titration) led to the conclusion that GdmCl (or urea) denatured proteins are devoid of elements of native secondary and tertiary structures (for review see [1]). The fact of the matter is that proteins thus denatured do contain some sequence-local and long-range residual structure ([1,2] and refs. therein). However, it has been argued that the presence of such residual structures in these chemically denatured proteins, are unlikely to affect significantly their random coil behavior. At least for two reasons, another denatured state that has drawn our attention is the heat denatured state of proteins. First is to find an answer to the question: Is denatured state of a protein induced by heat structurally identical to that obtained in GdmCl (or urea)? Second is to provide authentic protein stability parameters from differential scanning calorimetric (DSC) measurements in the absence of any chemical denaturants, and compare them with those obtained from GdmCl (or urea) denaturation.

More than fifty years ago, Bigelow [3] collected quantitative data for RNase-A measured by optical and hydrodynamic techniques. One of his significant conclusions was that, in terms of tertiary structure (exposure of Tyr), secondary structure and hydrodynamic volume, heat or acid-induced denatured state is less unfolded than the urea- and GdmCl-induced denatured state. (Later many studies from Bigelow’s lab followed in support of this conclusion (see, e.g., [4,5,6,7,8]). Furthermore, a survey of literature on the structural characterization of species involved in denaturations by heat and GdmCl, has revealed that, for a protein, the GdmCl-induced denatured state at 25°C is structurally more unfolded than the heat denatured state (see reviews [9,10,11,12,13,14,15,16,17,18]). The first most convincing evidence for the presence of native-like residual structure in the heat denatured state of proteins came from Tanford and associates [19] who observed another cooperative transition between heat-denatured (X) state and GdmCl denatured (D) state on adding GdmCl to already heat-denatured proteins. (Latter many studies supported this observation (see, e.g., [8,20,21,22]).) All these studies led to the conclusion that the heat denatured state of proteins are less unfolded than the GdmCl-induced denatured state. For the first time, the belief in this conclusion was shaken by Privalov and associates [23]. Their (i) differential scanning calorimetric (DSC) measurements of proteins in the presence and absence of GdmCl, (ii) isothermal titration calorimetric (ITC) measurements of GdmCl-induced denaturation of the native and heat-denatured proteins, and (iii) measurements of the constant-pressure heat capacity changes of proteins and those of protein groups (N- and C-termini, amino acid side chains and peptide bonds), led them to two definite conclusions. These are: (i) “…the values of enthalpy and entropy of their thermal denaturation are the same as those for GdmCl-induced denaturation if the letter process is properly corrected for salvation effect…”, and (ii) “… the correspondence of the heat capacity of denatured proteins with heat capacity expected for the unfolded polypeptide chain, which can be accurately calculated using the known heat capacities of amino acid residues, appear to be the one of the strongest criteria for completeness of unfolding…”. Hence, Privalov [23] remarked, “…on treating heat-denatured protein with GdmCl, the optical properties exhibit changes which were interpreted as an additional unfolding of the residual structure [19]… But a careful investigation … revealed that: (a) The observed changes of the optical parameters on the addition of GdmCl to heat-denatured protein cannot be interpreted within the framework of additional unfolding of the structure, since the observed changes are in the opposite direction and seem to be only a manifestation of salvation phenomena [24]”. A support to his argument came from hydrogen-exchange labeling in combination with NMR spectroscopy [25] and from the infrared spectroscopy showing evidence for lack of native-like structure in the thermally denatured RNase-A [26].

In order to resolve this issue of whether the additional transition of a physical property observed on the addition of GdmCl to the acid/heat denatured protein is due to a cooperative melting of residual structure or manifestation of salvation phenomena, we have measured changes in the far-UV CD (circular dichroism) of acid/heat denatured RNase-A, lysozyme and chymotrypsinogen on the addition of different concentration of GdmCl under the same experimental condition used by Anne et al. [19]. The observed sigmoidal curve of the far-UV CD signals (sensitive probe for measuring changes in secondary structure) of each protein was analyzed for Gibbs free energy change in the absence of the denaturant (Δ*G* ^0^_X→D_) associated with the process heat denatured (X) state ↔ GdmCl denatured (D) state. To confirm that this thermodynamic property represents the property of the protein alone and is not a manifestation of salvation effect, we measured urea-induced denaturation curves of these heat denatured proteins under the same experimental condition in which GdmCl-induced denaturation was carried out. An additional protein that we chose for this study was α-lactalbumin which undergoes reversible heat-induced and chemical-induced denaturations at pH 7.0, and its heat denatured state retains a large amount of secondary structure [27]. In this paper we report that (a) heat denatured proteins contain residual secondary structure, and GdmCl (or urea) induces a cooperative transition between X and D states, and (b) for each protein at a given pH and temperature, thermodynamic cycle connects quantities, Δ*G* ^0^_N→X_ (Gibbs free energy change associated with the thermal denaturation (native (N) state ↔ X state) in the absence of chemical denaturants, Δ*G* ^0^_X→D_ and Δ*G* ^0^_N→D_ (Gibbs free energy change associated with GdmCl (or urea)-induced denaturation (N state ↔ D state) in the absence of the chemical denaturant.

## Materials and Methods

Bovine pancreas ribonuclease-A (Type III A, lot 41K-7651), hen egg-white lysozyme (lot 111H-7010), bovine pancreasα-chymotrypsinogen-A (Type II, 16H-7075), and α-lactalbumin from bovine milk (Type I, lot 92H-7015) were purchased from Sigma Chemical Co. Analytical grade sodium cacodylate and EGTA (ethylene glycol-bis (β-aminethylether) N, N, N’, N’-tetra acetic acid) were also purchased from Sigma Chemical Co. Urea and GdmCl were ultrapure grade samples purchased from ICN Biomedical, Inc. KCl, glycine and other chemicals were of reagent grade and were used without further purification.

Lysozyme, ribonuclease-A (RNase-A), α-chymotrypsinogen-A (ctg) and α-lactalbumin were dialyzed in cold (at ~ 4°C) against several changes of 0.1 M KCl solution (pH 7.0). Apo- α-lactalbumin (apo-La) was prepared by adding 4 mM EGTA to the solution of the holoprotein (with ca^2+^ bound). Protein concentration of stock solutions were determined experimentally using ɛ, the molar absorption coefficient (M^-1^ cm^-1^) values of 39000 at 280 nm for lysozyme [28], 9800 at 277.5 nm for RNase-A [29], 29210 at 280 nm for apo-La [30], and 50562 at 280 nm for ctg [31]. We have used known values of refractive index to determine concentrations of stock solutions of GdmCl and urea [32].

The buffers used throughout the denaturation studies were 0.1 M KCl-HCl buffer (pH 2.0) for lysozyme and ctg, 0.1 M glycine-HCl buffer (pH 2.2) containing 0.1 M KCl for RNase-A, and 0.05 M cacodylic acid buffer (pH 7.0) containing 0.1 M NaCl and 4 mM EGTA for apo-La.

Circular dichroism (CD) measurements were carried out in Jasco Spectropolarimeter (model J-1500) equipped with the Peltier-type temperature controller. Protein concentrations used for CD measurements were in the range of 0.2–0.4 mg/ml, and 0.1 cm path length cell was used for the measurements in the far-UV region. CD instrument was routinely calibrated with d-10-camphorsulfonic acid. For isothermal measurements, a scan rate of 100 nm/min with a band width of 1 nm and response time of 1 sec was always used, and 4–6 spectra were accumulated. However, during measurements of thermal denaturation curves, we have used response time of 4 sec, band width of 2 nm and step resolution of 0.1°C. Results of all CD measurements are expressed as mean residue ellipticity ([*θ*]_λ_) in deg cm^2^ dmol^-1^ at a given wavelength, λ (nm) using the relation,

[θ]λ = θλM0/10cl (1)

where θ_λ_ is the observed ellipticity in millidegrees at wavelength λ,*M*_0_ is the mean residue weight of the protein, *c* is the protein concentration (mg/cm^3^), and *l* is the path length (cm). It should be noted that each observed θ_λ_ of the protein was corrected for the contribution of the solvent.

Heat-induced denaturation curves of proteins were obtained at three different scan rates, namely, 0.5, 1 and 1.5°C per minute. It has been observed that the thermodynamic parameters are independent of these scan rates. This observation indicates that the denaturation process does not occur as a kinetically controlled process, and each scan rate provided an adequate time for equilibration. However, we have obtained denaturation curves at a heating rate of 1°C in the temperature range 20–85°C. Reversibility of the heat-induced denaturation was checked by measuring the CD spectrum of the renatured protein after heating, and comparing it with that of the unheated protein sample. For this purpose, (i) CD spectrum of the native protein (unheated sample) was measured at 20°C, (ii) the protein sample was heated and the scan was terminated at 85°C, (iii) the denatured protein sample was cooled immediately to 20°C, and (iv) the CD spectrum of the renatured protein was determined at 20°C. Reversibility of thermal denaturation was confirmed by the identical CD spectra of the protein before heating and after cooling of the denatured protein solution.

Details of the preparation of protein solutions for isothermal urea- and GdmCl-induced denaturation experiments and those of renaturation experiments were reported earlier [33]. Reversibility of the GdmCl (or urea) denaturation was completely established as revealed by the identical CD value at a given denaturant concentration during denaturation and renaturation experiments. It should be noted that for the GdmCl- and urea-induced denaturation experiments at 25°C, required amounts of protein, denaturant and buffer solutions, were mixed at room temperature and kept there for equilibration.

## Results

### Isothermal GdmCl-induced and urea-induced denaturations of proteins

**Fig 1:**
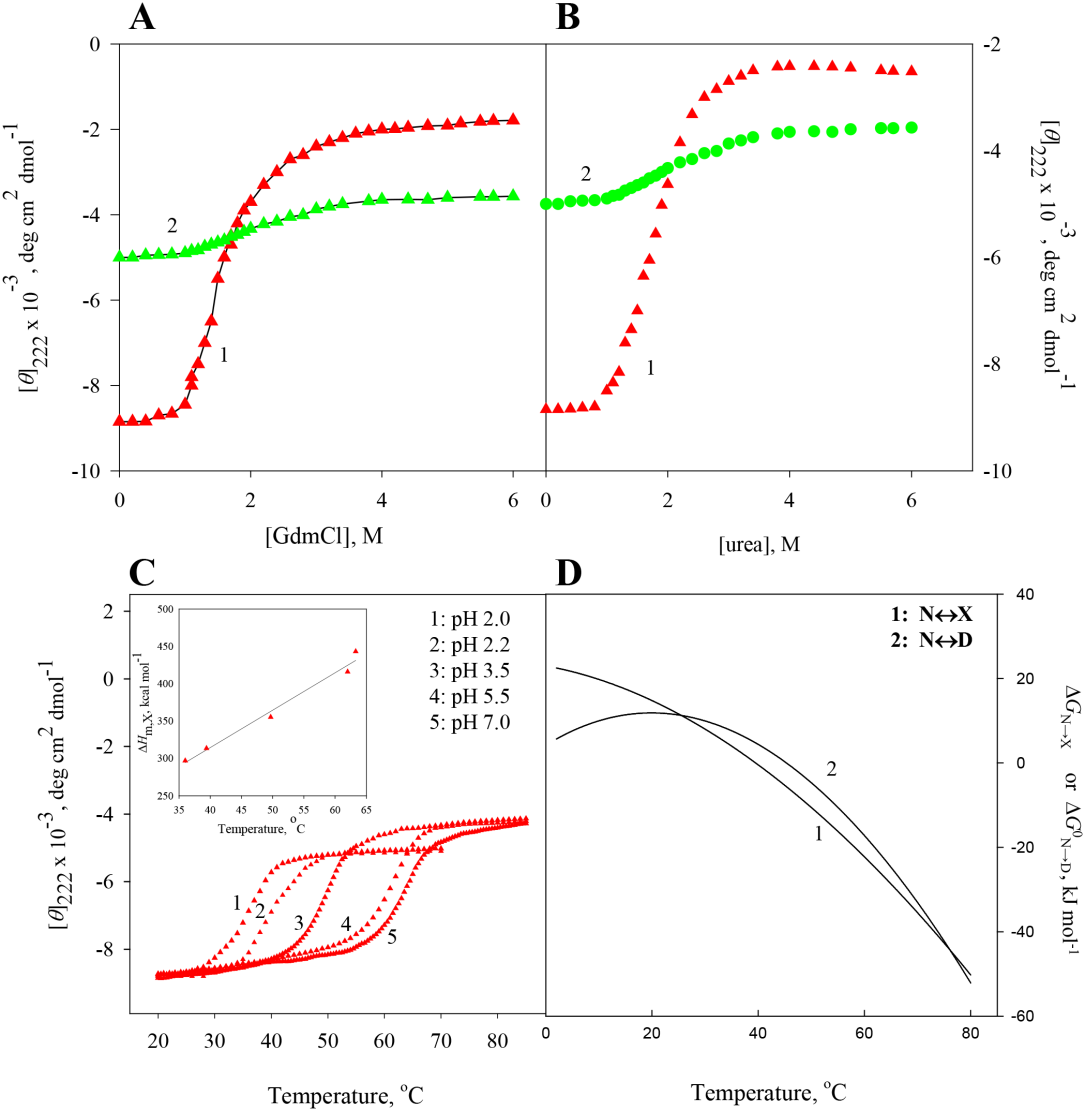
Denaturation of RNase-A at pH 2.2. **(A)** GdmCl-induced transition between N and D states at 25°C (curve 1) and transition between X and D states at 56°C (curve 2). **(B)** Urea-induced transition between N and D states at 25°C (curve 1), and transition between X and D states at 56°C (curve 2). **(C)** Thermal denaturation of RNase-A in the absence of the denaturant at different pH values. The inset shows plot of Δ*H*_m,x_ versus *T*_m,x_. **(D)** Curve 1 was drawn using Eq (5) with values of Δ*H*_m,x_ = 288 kJ mol^-1^, *T*_m,x_ = 39.5°C, Δ*C*_p,x_ = 5.14 kJ mol^-1^ K^-1^ given in Table 2, and curve 2 was drawn using Eq (6) with values of Δ*H*_m,D_ = 293 kJ mol^-1^, *T*_m,D_ = 45.3°C, Δ*C*_p,D_ = 11.07 kJ mol^-1^ K^-1^ given in Table 2.

**Fig 2:**
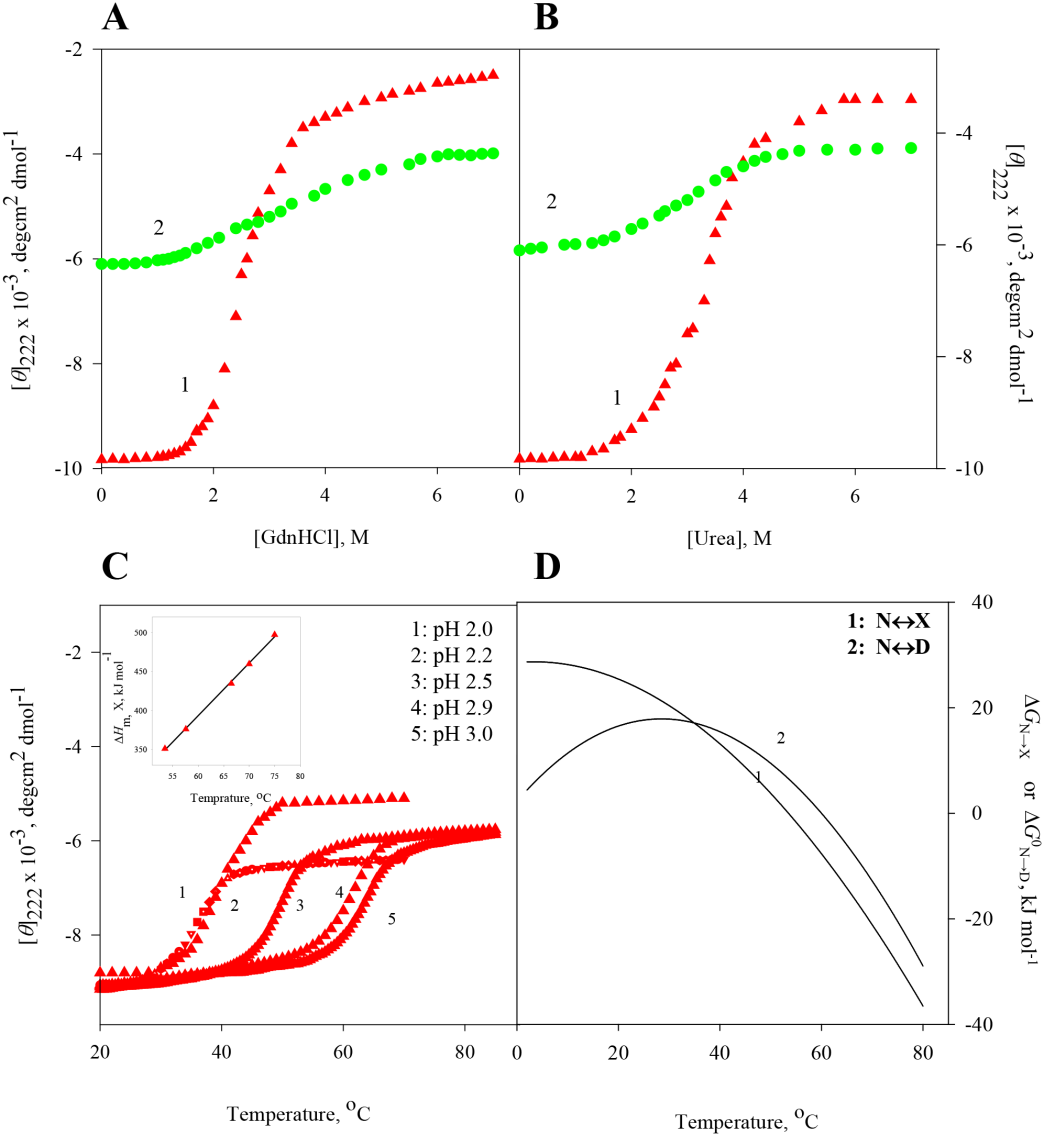
Denaturation of lysozyme at pH 2.0. **(A)** GdmCl-induced transition between N and D states at 25°C (curve 1) and transition between X and D states at 65°C (curve 2). **(B)** Urea-induced transition between N and D states at 25°C (curve 1), and transition between X and D states at 65°C (curve 2). **(C)** Thermal denaturation of lysozyme in the absence of the denaturant at different pH values. The inset shows plot of Δ*H*_m,x_ versus *T*_m,x_. **(D)** Curve 1 was drawn using Eq (5) with values of Δ*H*_m,x_ = 364 kJ mol^-1^, *T*_m,x_ = 53.5°C, Δ*C*_p,x_ = 6.69 kJ mol^-1^ K^-1^ given in Table 2, and curve 2 was drawn using Eq (6) with values of Δ*H*_m,D_ = 372 kJ mol^-1^, *T*_m,D_ = 60.0°C, Δ*C*_p,D_ = 11.24 kJ mol^-1^ K^-1^ given in Table 2.

**Fig 3:**
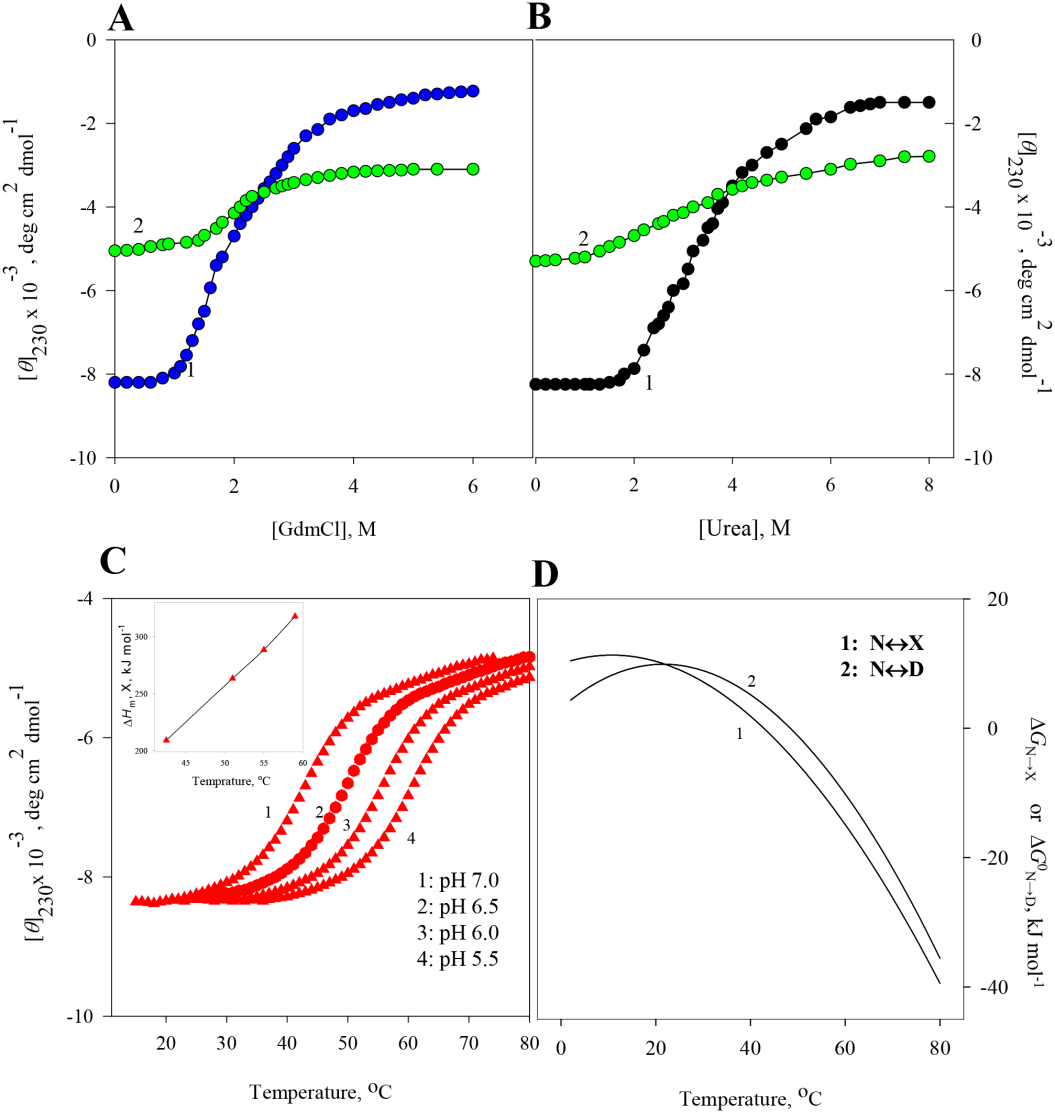
Denaturation of apo-La at pH 7.0. **(A)** GdmCl-induced transition between N and D states at 25°C (curve 1) and transition between X and D states at 60°C (curve 2). **(B)**. Urea-induced transition between N and D states at 25°C (curve 1), and transition between X and D states at 60°C (curve 2). **(C)** Thermal denaturation of apo-La in the absence of the denaturant at different pH values. The inset shows plot of Δ*H*_m,x_ versus *T*_m,x_. **(D)** Curve 1 was drawn using Eq (5) with values of Δ*H*_m,x_ = 219 kJ mol^-1^, *T*_m,x_ = 42.8°C, Δ*C*_p,x_ = 6.48 kJ mol^-1^ K^-1^ given in Table 2, and curve 2 was drawn using Eq (6) with values of Δ*H*_m,D_ = 234 kJ mol^-1^, *T*_m,D_ = 48.4°C, Δ*C*_p,D_ = 8.36 kJ mol^-1^ K^-1^ given in Table 2.

**Fig 4:**
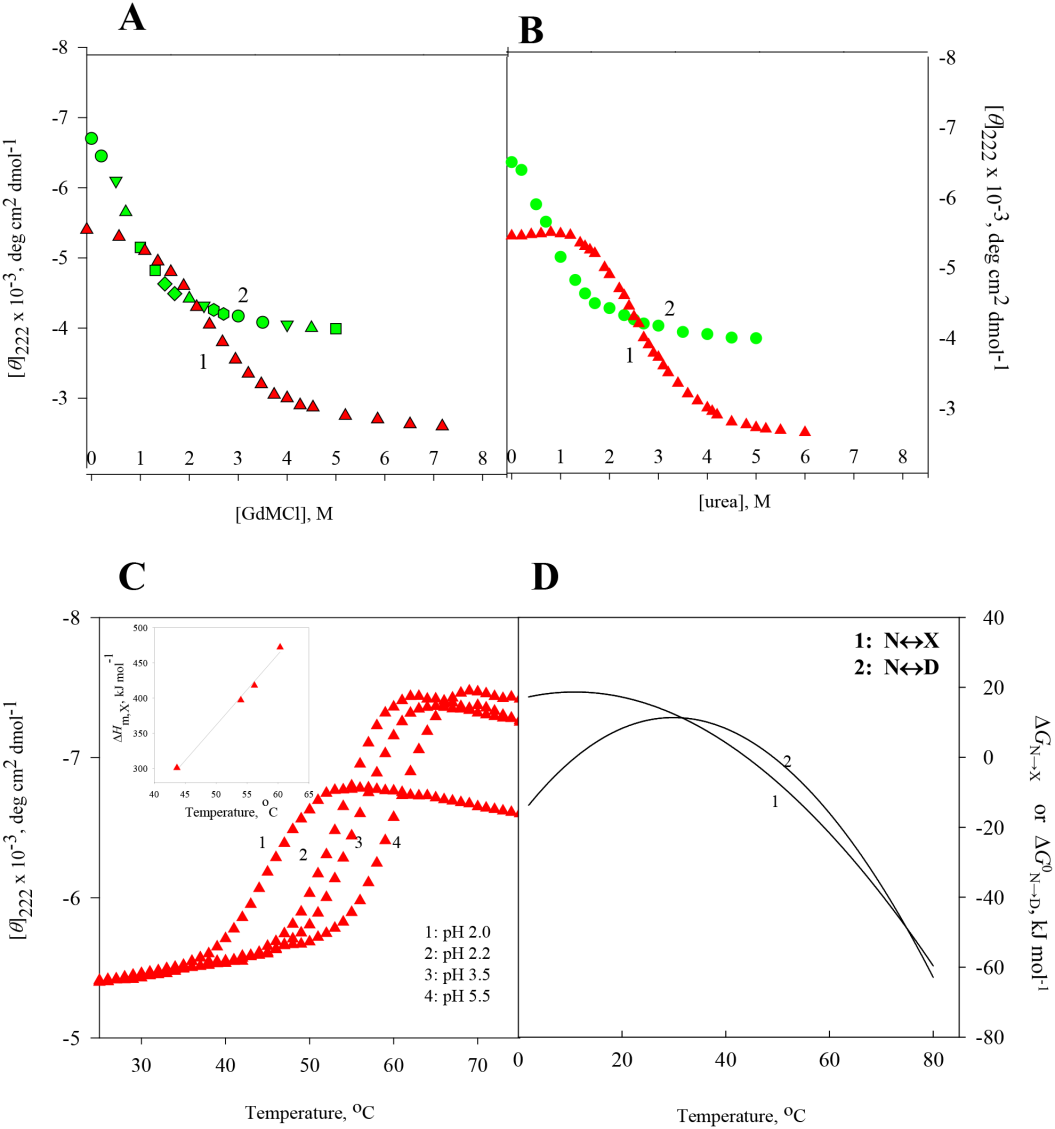
Denaturation of ctg at pH 2.0 **(A)** GdmCl-induced transition between N and D states at 25°C (curve 1) and transition between X and D states at 60°C (curve 2). **(B)** Urea-induced transition between N and D states at 25°C (curve 1), and transition between X and D states at 60°C (curve 2). **(C)** Thermal denaturation of ctg in the absence of the denaturant at different pH values. The inset shows plot of Δ*H*_m,x_ versus *T*_m,x_. **(D)** Curve 1 was drawn using Eq (5) with values of Δ*H*_m,x_ = 351 kJ mol^-1^, *T*_m,x_ = 44.0°C, Δ*C*_p,x_ = 10.03 kJ mol^-1^ K^-1^ given in Table 2, and curve 2 was drawn using Eq (6) with values of Δ*H*_m,D_ = 376 kJ mol^-1^, *T*_m,D_ = 49.2°C, Δ*C*_p,D_ = 18.90 kJ mol^-1^ K^-1^ given in Table 2.

At constant temperatures and pH values, the far-UV (250–205 nm) CD spectra of RNase-A, lysozyme, apo-LA and ctg in the absence and presence of various concentrations of GdmCl and urea were measured. The raw CD data which had photomultiplier voltage of less than 800 volt, were converted to concentration independent parameter, [*θ*]_λ_, using Eq (1). S1–S8 Figs show the far-UV CD spectra of all the four proteins. For each protein, values of [*θ*]_λ_, a probe to measure secondary structure [34,35] were plotted as a function of [GdmCl] and [urea], where square bracket represents molar concentration of the denaturant. Panels A and B of Figs 1–4 show respectively isothermal GdmCl-induced and urea-induced denaturation transitions between N (native) and D (GdmCl- (or urea-) induced denatured) states at 25°C (curve 1). These panels also show isothermal GdmCl-induced and urea-induced denaturation transitions between X (heat-induced denatured) state and D (GdmCl- (or urea-) induced denatured) state at a temperature at which X state exists (curve 2). It should be noted that the transition between X state and D state was measured at 65°C in the case of lysozyme, 56°C in the case of RNase-A, and 60°C in cases of ctg and apo-La. Except for GdmCl-induced denaturation of ctg, all transition curves of proteins were found reversible in the entire concentration range of GdmCl and urea. Although addition of GdmCl at concentrations below 1.7 M to ctg solution leads to protein precipitation, GdmCl-induces a reversible denaturation above 1.7 M at both 25°C and 60°C.

### Analysis of the isothermal transition curves

Assuming that the chemical denaturant-induced transition (i (initial) state ↔ f (final) state) follows a two-state mechanism, the entire ([*θ*][d] data of each transition curve for all the proteins were analyzed for Δ*G* ^0^_i→f_ and *m*_i→f_ using a nonlinear least-squares method according to the relation [36],

$$y([\text{d}])=\frac{y_{\text{i}}([\text{d}])+y_{\text{f}}([\text{d}])\text{ Exp}[-(\Delta G^{0}{}_{\text{i}\to \text{f}}+m_{\text{i}\to \text{f}}[\text{d}])/RT]}{1+\text{Exp}[-(\Delta G^{0}{}_{i\to f}+m_{i\to f}[\text{d}])/RT]}$$ (2)

where *y*([d]) is the observed optical property at [d], the molar concentration of the chemical denaturant, and *y*_i_([d]) and *y*_f_([d]) are respectively optical properties of the initial and final states of protein molecules under the same experimental conditions in which *y*([d]) was measured. In this equation Δ*G* ^0^_i→f_ is the Gibbs free energy change (Δ*G*_i→f_) in the absence of the denaturant, *m*_i→f_ is the slope (∂Δ*G*_i→f_/∂[d]), *R* is the universal gas constant, and *T* is the temperature in Kelvin. Here in this equation, i (initial) state represents N state for the process N state ↔ D state and X state for the process X state ↔ D state, and f (final) state represents GdmCl- (or urea-) induced D state. In fitting denaturation curves it has been assumed that the dependencies of *y*_i_ and *y*_f_ on [d] are linear (i.e., *y*_i_([d]) = *a*_i_ + *b*_i_[d] and *y*_f_([d]) = *a*_f_ + *b*_f_[d], where *a* and *b* are [d] independent constants, and subscripts i and f with these constants represent values for the pre-transition and post-transition base lines, respectively). Another assumption in the analysis was that Δ*G*_i→f_ is linear in [d]. Values of *C*_m_, the midpoint of transition curves, for N state ↔ D state and X state ↔ D state may be calculated from *C*_m,N→D_ = Δ*G* ^0^_N→D_/*m*_N→D_ and *C*_m,X→D_ = Δ*G* ^0^_X→D_/*m*_X→D_, respectively. Values of Δ*G* ^0^_N→D_,*m*_N→D_, Δ*G* ^0^_X→D_, and *m*_x→D_, are given in Table 1. These values are in excellent agreement with those reported earlier [13,37].

**Table 1:** Thermodynamic parameters for GdmCl-induced and urea induced denaturations of RNase-A (pH 2.2), lysozyme (pH 2.0), apo-LA (pH 7.0) and ctg (pH 2.0)^a^.

Denaturant	N state ↔ D state^b^		X state ↔ D state^c^	
	Δ*G* ^0^_N→D_kJ mol^-1^	*m*_N→D_ kJ mol^-1^M^-1^	Δ*G* ^0^_X→D_ kJ mol^-1^	*m*_X→D_kJ mol^-1^M^-1^
**RNase-A**				
GdmCl	11.37±0.75	-8.11±0.40	6.64±0.60	-5.18±0.12
Urea	11.58±0.50	-6.90±0.33	7.11±0.46	-4.26±0.38
**Lysozyme**				
GdmCl	17.35±0.33	-7.44±0.50	8.99±0.33	-3.72±0.25
Urea	17.56±0.50	-5.48±0.37	9.24±0.33	-3.43±0.29
**Apo-La**				
GdmCl	10.12±0.33	-4.83±0.25	6.88±0.33	-5.04±0.29
Urea	9.20±0.71	-3.13±0.21	5.43±0.46	-2.67±0.29
**Ctg** ^d^ ^,^ ^e^				
Urea	11.08±0.84	-4.56±0.33	6.85±0.33	-4.60±0.04
	(10.28±0.96)	(-3.93±0.33)	nd^f^	nd

^a^A '±' with each parameter indicates mean error from three independent measurements.

^b^The transition N state ↔ D state was measured at 25°C.

^c^The transition X state ↔ D state was measured at 56°C for RNase-A, 65°C for lysozyme, and 60°C for both apo-La and ctg.

^d^GdmCl-induced denaturations are irreversible.

^e^Values in parentheses are obtained from denaturation curve monitored by [*θ*]_260_ measurement at 25°C.

^f^Not determined.

### Heat-induced denaturation (N state ↔ X state) of proteins

Thermal denaturation curves of RNase-A, lysozyme, apo-LA and ctg in the absence of GdmCl and urea were measured at different pH values by monitoring changes in [*θ*] in the temperature range 20–85°C. The reversibility of thermal denaturation was completely established as revealed by the identical CD spectra before and after heating the sample solutions. Panel C of Figs 1–4 shows respectively heat-induced transitions of RNase-A, lysozyme, apo-La and ctg at different pH values. Each heat-induced (N state ↔ X state) denaturation curve of a protein in the absence of chemical denaturant at a given pH was analyzed for *T*_m,X_ (midpoint of thermal denaturation) and Δ*H*_m,X_ (enthalpy change at *T*_m,X_) using a nonlinear least-squares method according to the relation [36],

$$\;y(T)=\frac{y_{\text{N}}(T)+y_{\text{X}}(T)Exp[-\Delta H_{\text{m},\text{x}}/R(1/T-1/T_{\text{m},\text{x}})]}{1+\text{Exp}[-\Delta H_{\text{m},\text{x}}/R(1/T-1/T_{\text{m},\text{x}})]}$$ (3)

where *y*(*T*) is the experimentally observed optical property of the protein at temperature *T* K, *y*_N_(*T*) and *y*_X_(*T*) are the optical properties of the native and denatured protein molecules at *T* K, respectively, and *R* is the gas constant. In the analysis of the transition curve, it was assumed that a parabolic function describes the dependence of the optical properties of the native and denatured protein molecules (i.e., *y*_N_(*T*) = *a*_N_ + *b*_N_ *T* + *c*_N_ *T* ^2^ and *y*_X_(*T*) = *a*_X_ + *b*_X_ *T* + *c*_X_ *T* ^2^, where *a*_N,_ *b*_N,_ *c*_N,_ *a*_X,_ *b*_X_ and *c*_X_ are temperature independent coefficients) [38]. Values of Δ*H*_m,X_ and *T*_m,X_ for all proteins are given in Table 2.

**Table 2:** Thermodynamic parameters associated with heat-induced and urea (or GdmCl)-induced denaturations of proteins in the absence of the chemical denaturants^a^.

Proteins	N state ↔ X state				N state ↔ D state^b^			
	*T*_m,X_(°C)	Δ*H*_m, X_ (kJ mol^-1^)	Δ*C*_p,X_ (kJ mol^-1^K^-1^)	Δ*G* ^0^_N↔X_ (kJ mol^-1^)	*T* ^0^_m,D_ (°C)	Δ*H* ^0^_m,D_ (kJ mol^-1^)	Δ*C* ^0^_p,D_ (kJ mol^-1^K^-1^)	Δ*G* ^0^_N↔D_ (kJ mol^-1^)
RNase-A (pH 2.2)	39.5 ± 0.3	288 ± 12	5.14 ± 0.46	11.60 ± 0.84	45.3 ± 0.3 (44.9 ± 0.4)	293 ± 10 (293 ± 8)	11.07 ± 0.84 (10.45± 0.83)	11.36 (11.67)
Lysozyme (pH 2.0)	53.5 ± 0.3	364 ± 21	6.69 ± 0.29	23.19 ± 2.09	60.0 ± 0.5 (59.3 ± 0.5)	372 ± 11 (376 ± 9)	11.24 ±0.84 (11.33 ±0.79)	17.65 (18.02)
Apo-La (pH 7.0)	42.8 ± 0.1	219 ± 8	6.48 ± 0.21	9.03 ± 1.67	48.4 ± 0.3 (49.3 ± 0.3)	234 ± 21 (230 ± 8)	8.36 ±0.86 (8.99± 0.88)	9.73 (8.89)
Ctg^c^ (pH 2.0)	44.0 ± 0.3 [39.7± 0.3]	351 ± 17 [355± 12]	10.03 ± 1.25	13.79 ± 1.25 [15.20 ± 1.25]	49.2 ± 0.3	376 ± 13	18.90 ±0.88	10.61

^a^A ± with each parameter is the mean deviation from the mean of triplicate measurements.

^b^Taken from Singh et al. (2008). Superscript, "0" represents the fact that these values are in the absence of urea and GdmCl (values in parenthesis).

^c^GdmCl-induced denaturation is not only irreversible but protein aggregation is also observed in lower denaturant concentration range (see text). Values given in square brackets are obtained from thermal denaturation curve monitored by [*θ*]_260_ measurements.

Value of the constant-pressure heat capacity change (Δ*C*_p,X_) was determined from the slope of the linear plots of Δ*H*_m,X_ versus *T*_m,X_ (see inset in panel C of Figs 1–4) according to the relation,

$$\Delta Cp,x\;=\;\left(\partial \Delta Hm,\frac{x}{\partial Tm,x}\right)p$$ (4)

Δ*C*_p,X_ values of all proteins are shown in Table 2. It should be noted that this analysis assumes that Δ*C*_p,x_ is independent of temperature. Using value of ΔH_m,X_, *T*_m,X_ and Δ*C*_p,X_, the change in Gibbs free energy (Δ*G*_N→X_(*T*)) can be calculated at any temperature *T* K using Gibbs-Helmholtz equation,

$$\Delta G_{\text{N}\to \text{X}}(T)=\Delta H_{\text{m},\text{X}}\left(\frac{T_{\text{m},\text{X}}-T}{T_{\text{m},\text{X}}}\right)-\Delta C_{\text{p},\text{X}}\left[(T_{\text{m},\text{X}}-T)+T\text{ln}\left(\frac{T}{T_{\text{m},\text{X}}}\right)\right]$$ (5)

where *T*_m,X_ is midpoint of thermal denaturation and Δ*H*_m,X_ is the enthalpy change at *T*_m_ for N state ↔ X state transition. Table 2 shows the values Δ*G* ^0^_N→X_ (value of Δ*G*_N→X_ at 25°C) of all proteins. Table 2 also contains values of thermodynamic parameters (ΔH^0^_m,D_, *T* ^0^_m,D_, Δ*C*_p,D_, and Δ*G* ^0^_N→D_) associated with N state ↔ D state in the absence of strong chemical denaturants, GdmCl and urea [27].

It should be noted that, unlike heat-induced denaturation of RNase-A, lysozyme and apo-La, the change in ellipticity of ctg is in opposite direction suggesting heat induces more secondary structure in this protein. This observation is in agreement with that reported earlier [17]. To know the effect of temperature on the tertiary structure of ctg, we have measured the heat-induced transition curve by following changes in the molar elipticity, [*θ*], at 260 nm, a wavelength which reflects the global disruption of tertiary structure. This transition curve at pH 2.0 (curve not shown) was analyzed for Δ*H*_m,X_ and *T*_m,X_. Values of these parameters are given in Table 2 (see values in square brackets). It is seen in this table that values of these thermodynamic quantities obtained from [*θ*]_222_ and [*θ*]_260_ measurements are in excellent agreement.

To check whether [*θ*]_260_ measures global disruption of tertiary structure of ctg, we measured urea-induced denaturation of the protein by following changes in [*θ*]_260_ at pH 2.0 and 25°C. The denaturation curve (not shown here) was analyzed for Δ*G* ^0^_N→D_ and *m*_N→D_, using Eq (2), and these values are given in parenthesis in Table 1. An agreement of these values of Δ*G* ^0^_N→D_ and *m*_N→D_ with those obtained from the denaturation curve of [*θ*]_222_ suggests that [*θ*]_260_ is really a measure of global change in the tertiary structure of ctg.

## Discussion

DSC can be used successfully for the direct estimation of thermodynamic parameters (Δ*H*_m_ and Δ*C*_p_) only when there is a good energetic difference in the two end states of the denaturation equilibrium, e.g., X state ↔ D state. If there is not a good enthalpy difference between two structurally different states X and D, an endothermic peak is not expected to be observed by DSC scan for the transition X state ↔ D state. In fact, it has been reported earlier [23] that calorimetric Δ*H*_m,X_ and ΔH_m,d_ are not significantly different for proteins. Therefore, we needed a probe that can detect the subtle changes in the structural characteristics during transition between X and D states. The far-UV CD serves an excellent probe for characterizing such states for a number of reasons. (i) CD spectrum provides not only qualitative and quantitative information about protein secondary structure but it is also a very sensitive probe to monitor the unfolding and refolding of protein secondary structure either at equilibrium, or kinetically. (ii) Even if there are high concentrations of denaturants, the protein conformational stability can still be monitored by CD spectra. (iii) Unlike DSC measurements, equilibrium constant (Gibbs free energy change) is directly measured as a function of temperature by CD temperature scanning, and all derived thermodynamic parameters (Δ*H*, Δ*S* and Δ*C*_p_) of proteins are obtained from differentiation of the van't Hoff equation [38,39]. Since for each protein, the far-UV spectrum of X state is different from spectra of N and D states, we therefore measured Δ*G* values associated with N state ↔ D state, N state ↔ X state and X state ↔ D state of four proteins, namely, RNase-A, lysozyme, apo-La and ctg using the far-UV CD.

Since measurements of Δ*G* is possible only when a protein operates under certain constraints, a few comments are therefore necessary. (a) To estimate Δ*G* ^0^_N→D_, Δ*G* ^0^_X→D_, Δ*G*_N→X_ from denaturation curves, a two-state mechanism has been assumed under all conditions. This is indeed true for the heat-induced denaturation [23,40] and chemical-induced denaturation [17,41,42,43] of all the proteins studied here. Furthermore, our results shown in Tables 1 and 2 support that the urea-induced and heat-induced denaturations of ctg follow a two-state mechanism. The reasons for saying this are that (i) Δ*G* ^0^_N→D_ values obtained from urea-induced denaturation curves of [*θ*]_222_ and [*θ*]_260_ are in excellent agreement (see columns 2 and 3 of Table 1), and (ii) thermodynamic quantities obtained from thermal denaturation curves of [*θ*]_222_ and [*θ*]_260_ are in excellent agreement (see columns 2,3 and 5 of Table 2). (b) The linear extrapolation method (i.e., Δ*G*_i→f_ is a linear function of [GdmCl] and [urea]) is used for estimating the conformational stability (Δ*G* ^0^_N→D_ and Δ*G* ^0^_X→D_) of proteins. It should be noted that this method is based on solid thermodynamic ground [44] as well as experimental observations [33,43,45,46]. (c) Δ*G* ^0^_N→D_ and Δ*G* ^0^_X→D_ obtained from GdmCl-induced and urea-induced denaturations are the values which are yielded by the extrapolation of the plot of this thermodynamic quantity versus chemical denaturant concentration to 0 M. If the thermodynamic quantity, Δ*G* ^0^_N→D_ (or Δ*G* ^0^_X→D_) is identical from both GdmCl-induced and urea-induced denaturations, then it is the property of the protein alone and not of the protein and chemical denaturant. That is why we carried out both GdmCl-induced and urea-induced denaturations of the native and heat denatured states, i.e., N state ↔ D state and X state ↔ D state. It is seen in Table 1 that for each protein values of Δ*G* ^0^_N→D_ and Δ*G* ^0^_X→D_ derived from the GdmCl- and urea-induced denaturations are, within experimental errors, identical, suggesting that that Δ*G* ^0^_N→D_ and Δ*G* ^0^_X→D_ are the properties of the protein alone.

Calorimetric data indicated that thermally and chemically denatured states are not only energetically identical [23,47] but also, for a protein, the additional transition (X state ↔ D state) of the optical rotation [19] induced by GdmCl, is only a manifestation of solvation phenomena [23]. To show that the heat denatured state is thermodynamically different in terms of Gibbs free energy change, and this thermodynamic property is independent of the chemical denaturants, we (a) measured heat-induced and isothermal GdmCl (and urea)-induced denaturation curves of all proteins by following changes in the far-UV CD, (b) analyzed these denaturation curves for Δ*G* ^0^ (Δ*G* in the absence of chemical denaturant) values for N state ↔ X state, X state ↔ D state and N state ↔ D state at the same temperature and pH, and (c) employed these Δ*G* ^0^ values in a thermodynamic cycle. To estimate thermal stability parameters of proteins, we carried out thermal denaturation of RNase-A, lysozyme, apo-La and ctg at different pH values. All thermal transition curves were analyzed for Δ*H*_m,X_ and *T*_m,X_ according to Eq (3), and (Δ*H*_m,X_, *T*_m,X_) data obtained at different pH values were used to determine Δ*C*_p,X_. Values of these thermodynamic quantities for all proteins are given in Table 2, which are in excellent agreement with those reported earlier [23,40]. To see how Δ*G*_N→X_ varies with temperature, we estimated values of Δ*G*_N→X_(*T*) at different temperatures with the help of Eq (5) using values of Δ*H*_m,X_, *T*_m,X_ and Δ*C*_p,X_, and these values are shown in panel D of Figs 1–4. It is noteworthy that for all proteins thermal stability (Δ*G*_N→X_) increases with decreasing temperature, its maximum value is reached at temperature close to physiological or below. The existence of maxima is stipulated by Δ*C*_p,X_ of denaturation in Eq (5).

Table 1 shows values of Δ*G* ^0^_N→D_ obtained from the analysis of GdmCl-induced and urea-induced denaturation curves of all proteins (see panels A and B in Figs 1–4). These values are in excellent agreement with those reported earlier ([27] and refs therein). It can be seen from Tables 1 and 2 that Δ*G* ^0^_N→D_ with Δ*G* ^0^_N→X_ (i.e., at 25°C) of RNase-A and apo-La are, within experimental errors, identical whereas these are significantly different in cases of lysozyme and ctg. To see whether this is true at all temperatures we constructed stability curves for N state ↔ D state, using known values of Δ*H*_m,D_, *T*_m,D_ and Δ*C*_p,D_ (see Table 2) in Gibbs-Helmholtz equation (Eq 6),

$$\Delta G{{}^{\circ}}_{\text{N}\to \text{D}}(T)=\Delta H{{}^{\circ}}_{\text{m},\text{D}}\left(\frac{T{{}^{\circ}}_{\text{m},\text{D}}-T}{T{{}^{\circ}}_{\text{m},\text{D}}}\right)-\Delta C_{\text{p},\text{D}}\left[(T{{}^{\circ}}_{\text{m},\text{D}}-T)+T\text{ln}\left(\frac{T}{T{{}^{\circ}}_{\text{m},\text{D}}}\right)\right]$$ (6)

These results are shown in panel D of Figs 1–4. It is seen in these figures that Δ*G* ^0^_N→D_ (*T*) and Δ*G*_N→X_(*T*) are not the same at all temperatures. Therefore a coincidence or non-coincidence of ΔΔ*G* ^0^_N→D_ and Δ*G*_N→X_ values at a given temperature cannot be considered as proof for the absence or presence of residual structure in the heat denatured proteins [23]. Furthermore, it is also evident from Figs 1–4 (panel D) that *T*_max_ (temperature at which Gibbs energy change is maximum) is achieved earlier for N state ↔ D state than that for N state ↔ X state. This is an expected result because *T*_m,D_ and Δ*C*_p,D_ are larger than *T*_m,X_ and Δ*C*_p,X_ of proteins with similar Δ*H*_m,X_ and Δ*H*_m,D_ values.

**Fig 5:**
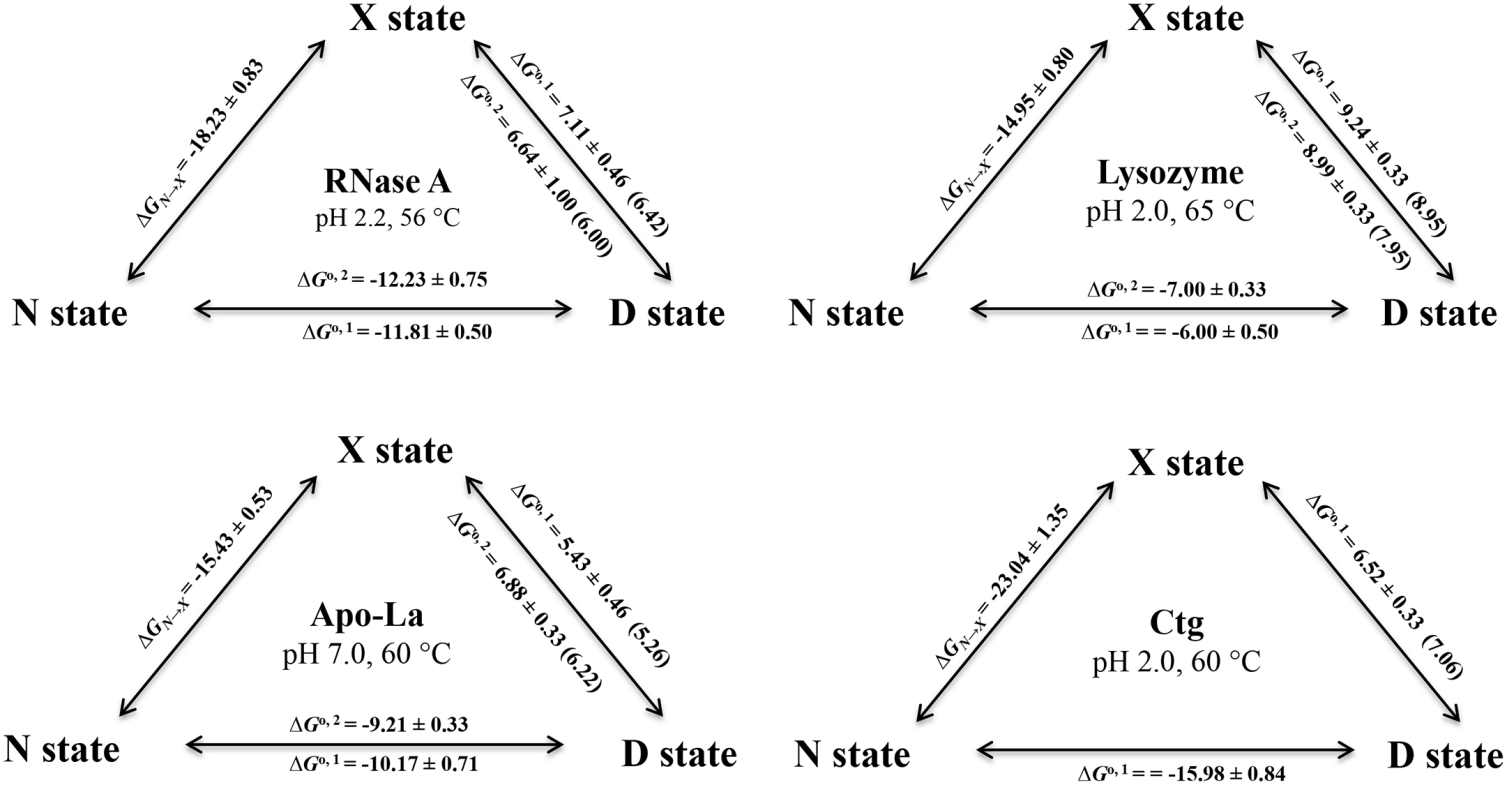
Gibbs free energy changes of proteins associated with the process N state ↔ X state ↔ D state in the absence of GdmCl and urea at indicated temperature and pH. Superscripts "1" and "2" in Δ*G* ^0,1^ and Δ*G* ^0,2^ represent values obtained from the urea- and GdmCl- induced denaturations, respectively. A value in the parenthesis is the value predicted from known values of Δ*G* ^0^_N→D_(*T*) and Δ*G*_N→X_(*T*) (i.e., Δ*G* ^0^_X→D_(*T*) = Δ*G* ^0^_N→D_(*T*)_—_Δ*G*_N→X_(*T*)).

Table 1 shows Δ*G* ^0^_X→D_ values obtained from the analysis of isothermal GdmCl- and urea induced denaturations of the already heat-denatured RNase-A, lysozyme, apo-La and ctg. It should be noted that no such data are available in literature for their comparison. A question arises: For a protein, is the heat denatured (X) state on the folding/unfolding pathway (i.e., N state ↔ X state ↔ D state)? To answer this question, we constructed a thermodynamic cycle. If X state is on the folding/unfolding pathway, then at a given pH and temperature (say at 25°C) the sum of Δ*G* ^0^_N→X_ and Δ*G* ^0^_X→D_ must be equal to Δ*G* ^0^_N→D_. **However**, we could not construct thermodynamic cycle at 25°C at which ΔΔ*G* ^0^_N→D_ is directly measured, and Δ*G* ^0^_N→X_ is obtained from stability curve drawn using Eq (5). The reason for this is that, due to experimental constraints, we could not estimate Δ*G* ^0^_X→D_ of proteins at 25°C, for there were not much variations in the Δ*G* ^0^_X→D_ values estimated above 56°C for RNase-A, 65°C for lysozyme and 60°C for apo-La and ctg. Therefore, we opted for extrapolations Δ*G*_N→X_(*T*) and Δ*G* ^0^_N→D_(*T*) to a temperature at which Δ*G* ^0^_X→D_ of the protein is directly measured. Values of Δ*G*_N→X_(*T*), Δ*G* ^0^_X→D_(*T*) and Δ*G* ^0^_N→D_(*T*) all at the same teperature are incorporated in the thermodynamic cycle for all the proteins (Fig 5). It is seen in Fig 5 that, for a protein, the sum of Δ*G* ^0^_X→D_(*T*) and Δ*G*_N→X_(*T*) is, within experimental errors, identical to Δ*G* ^0^_N→D_(*T*). The successful working of a thermodynamic cycle confirms that residual structures exist in thermally denatured RNase-A, lysozyme, apo-La and ctg, which can be cooperatively removed by addition of chemical denaturants, GdmCl and urea.

## Conclusions

We are sure of three things. (a) Thermally denatured proteins contain residual secondary structure that can be removed on addition of GdmCl and urea. (b) Transition X (heat denatured) state ↔ D (GdmCl or urea denatured) state is cooperative. (c) The heat denatured state exists on the folding pathway, N state ↔ D state.

## Supporting Information

S1 Fig GdmCl-induced denaturation of RNase-A in the presence of different concentrations of the denaturant at pH 2.2. The representative far-UV CD spectra of RNase-A at 25°C (A) and at 56°C (B).
(PPTX)

S2 Fig Urea-induced denaturation of RNase-A in the presence of different concentrations of the denaturant at pH 2.2. The representative far-UV CD spectra of RNase-A at 25°C (A) and at 56°C (B).
(PPTX)

S3 Fig GdmCl-induced denaturation of lysozyme in the presence of different concentrations of the denaturant at pH 2.0. The representative far-UV CD spectra at 25°C (A) and at 65°C (B).
(PPTX)

S4 Fig Urea-induced denaturation of lysozyme in the presence of different concentrations of the denaturant at pH 2.0. The representative far-UV CD spectra at 25°C (A) and at 65°C (B).
(PPTX)

S5 Fig GdmCl-induced denaturation of apo-La in the presence of different concentrations of the denaturant at pH 7.0. The representative far-UV CD spectra of apo-La at 25°C (A) and at 60°C (B).
(PPTX)

S6 Fig Urea-induced denaturation of apo-La in the presence of different concentrations of the denaturant at pH 7.0. The representative far-UV CD spectra of apo-La at 25°C (A) and at 60°C (B).
(PPTX)

S7 Fig GdmCl-induced denaturation of ctg in the presence of different concentrations of the denaturant at pH 2.0. The representative far-UV CD spectra of ctg at 25°C (A) and 60°C (B).
(PPTX)

S8 Fig Urea-induced denaturation of ctg in the presence of different concentrations of the denaturant at pH 2.0. The representative far-UV CD spectra of ctg at 25°C (A) and 60°C (B).
(PPTX)
