# Supplementary figures and images for: Cooperative Unfolding of Residual Structure in Heat Denatured Proteins by Urea and Guanidinium Chloride
Source: PLoS One. 2015 Jun 5;10(6):e0128740. doi: 10.1371/journal.pone.0128740 (PMC4457810; doi:10.1371/journal.pone.0128740)

## Slide 1
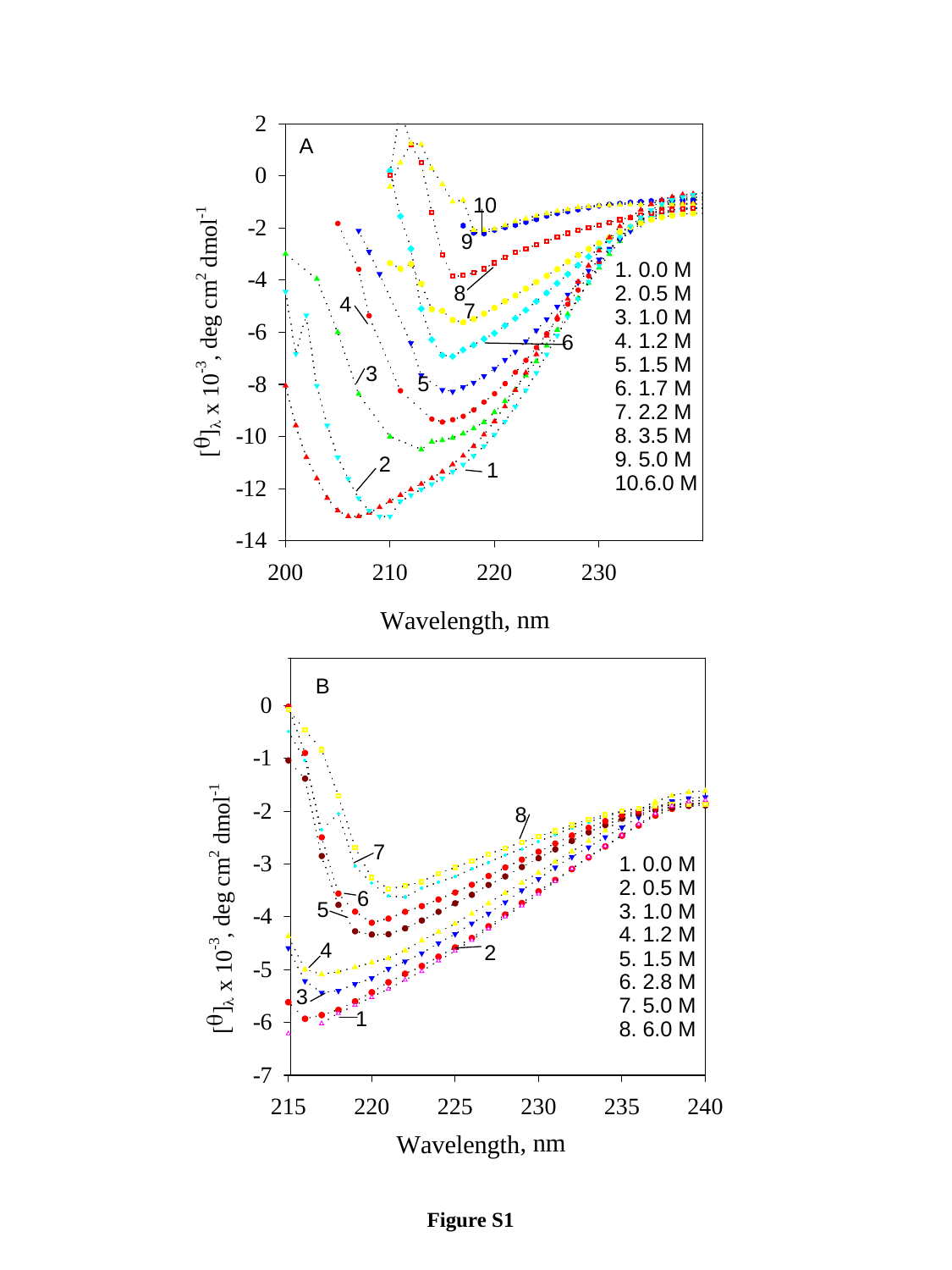

Figure S1

Supplement: S1 Fig — The representative far-UV CD spectra of RNase-A at 25°C (A) and at 56°C (B). (PPTX) [file pone.0128740.s001.pptx]

## Slide 1
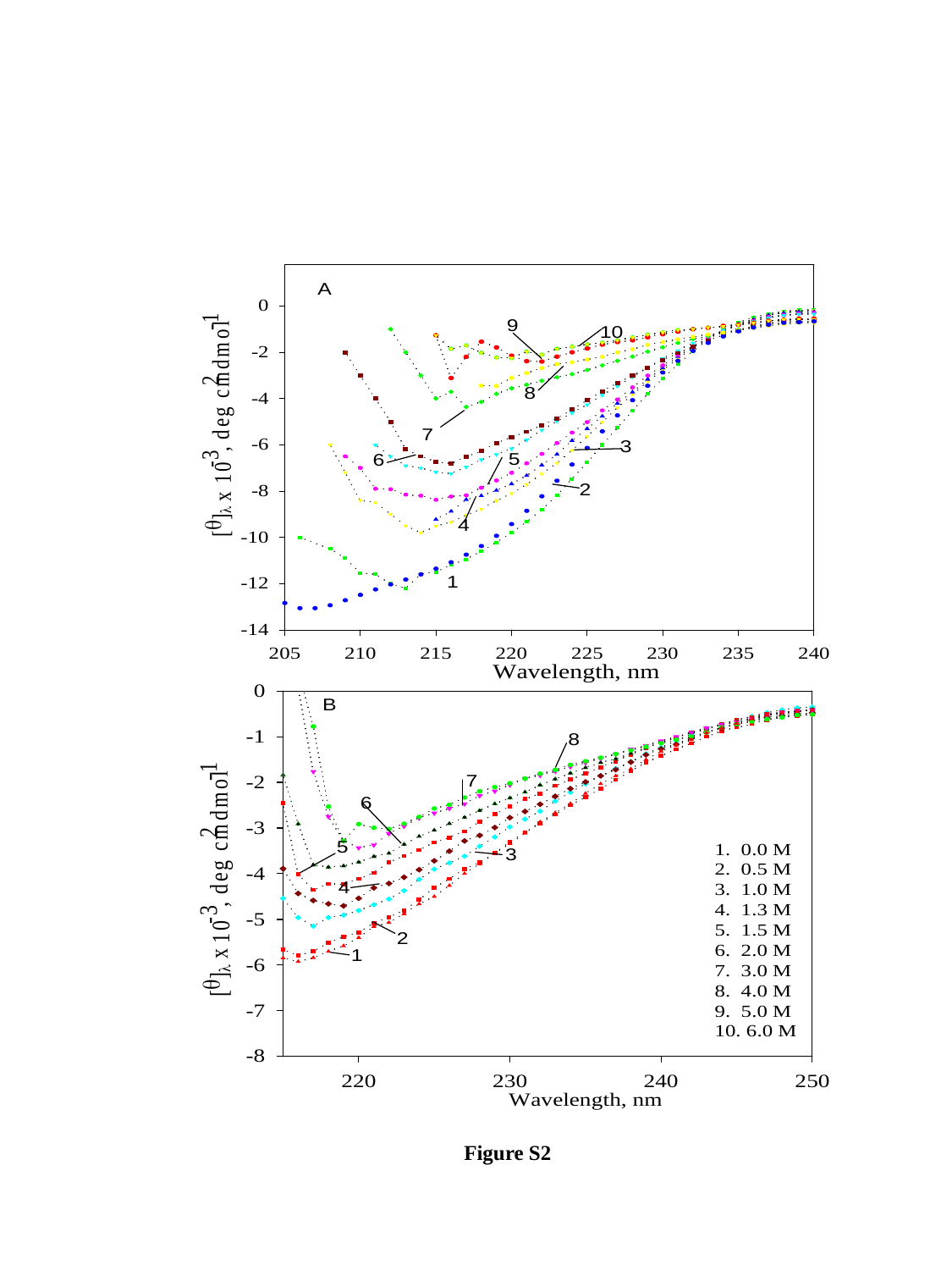

Figure S2

Supplement: S2 Fig — The representative far-UV CD spectra of RNase-A at 25°C (A) and at 56°C (B). (PPTX) [file pone.0128740.s002.pptx]

## Slide 1
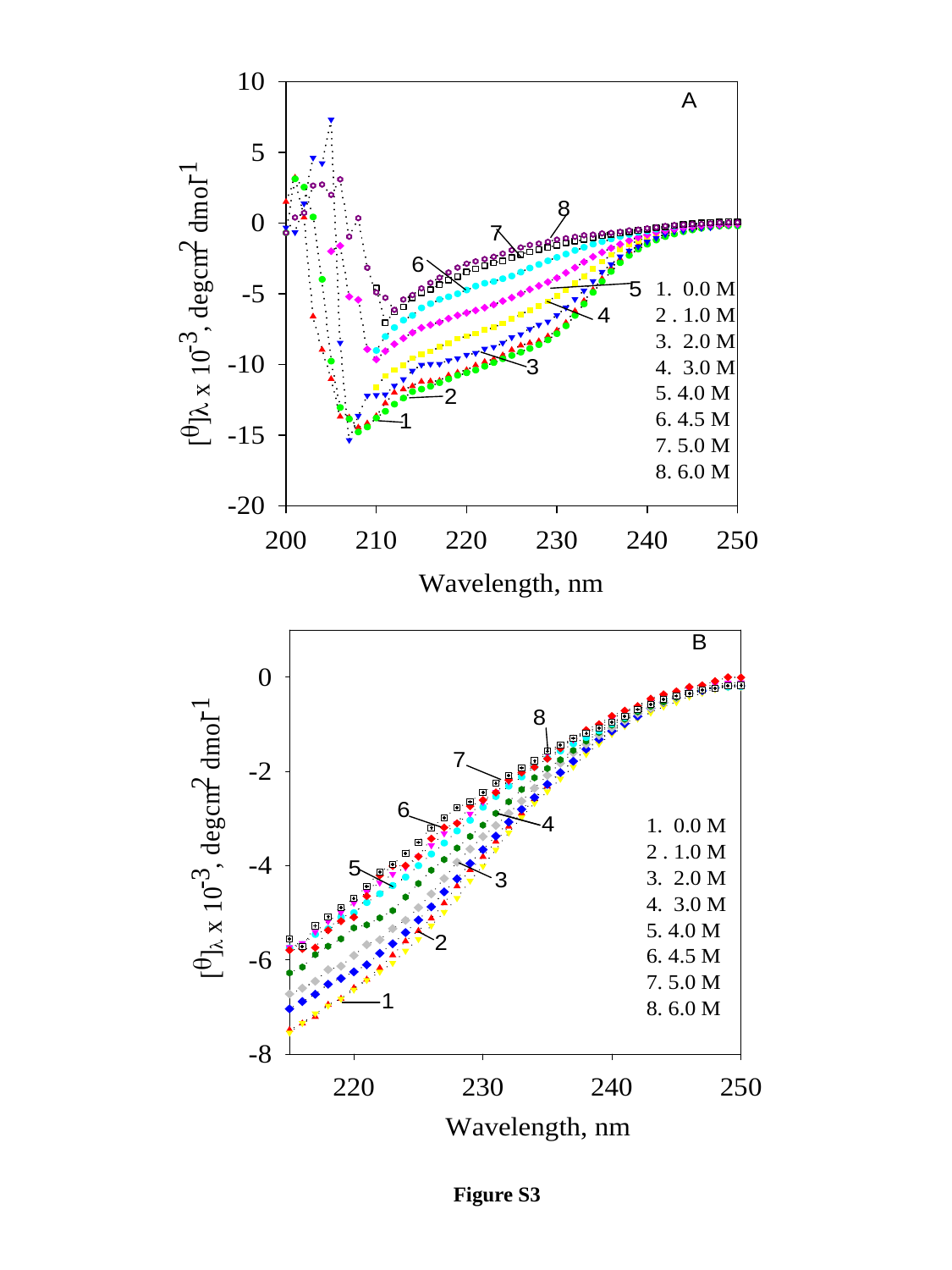

Figure S3

Supplement: S3 Fig — The representative far-UV CD spectra at 25°C (A) and at 65°C (B). (PPTX) [file pone.0128740.s003.pptx]

## Slide 1
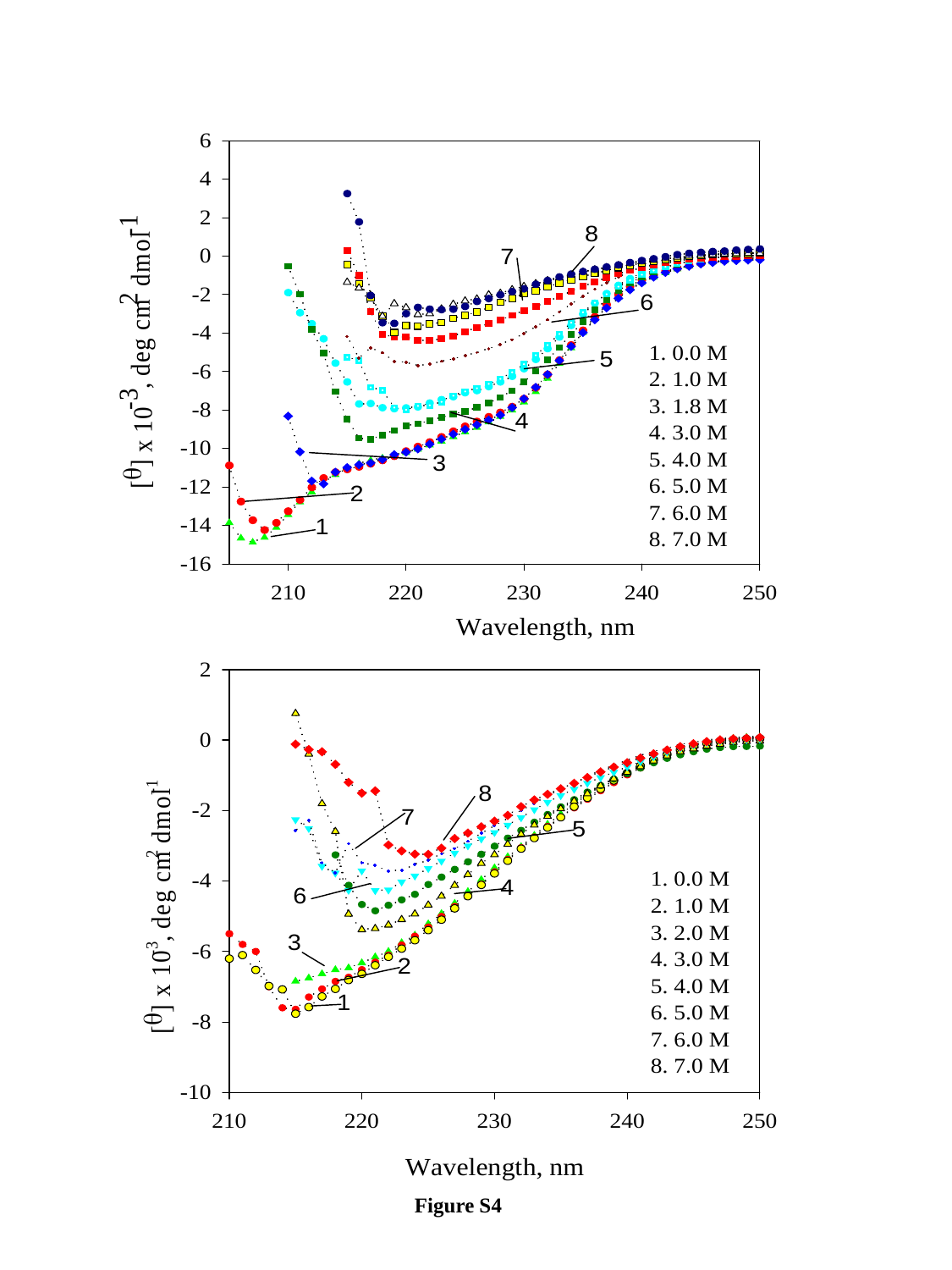

Figure S4

Supplement: S4 Fig — The representative far-UV CD spectra at 25°C (A) and at 65°C (B). (PPTX) [file pone.0128740.s004.pptx]

## Slide 1
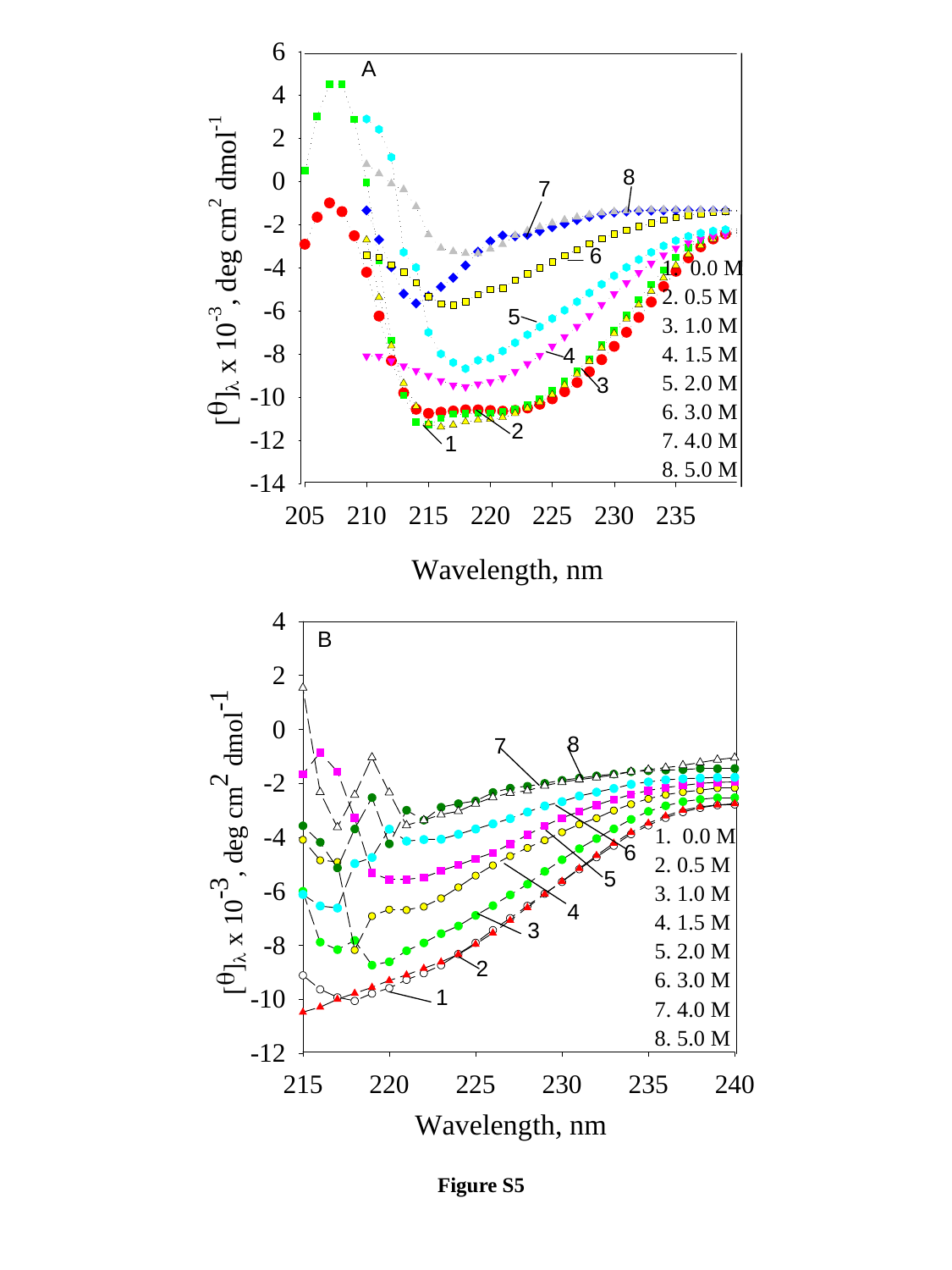

Figure S5

Supplement: S5 Fig — The representative far-UV CD spectra of apo-La at 25°C (A) and at 60°C (B). (PPTX) [file pone.0128740.s005.pptx]

## Slide 1
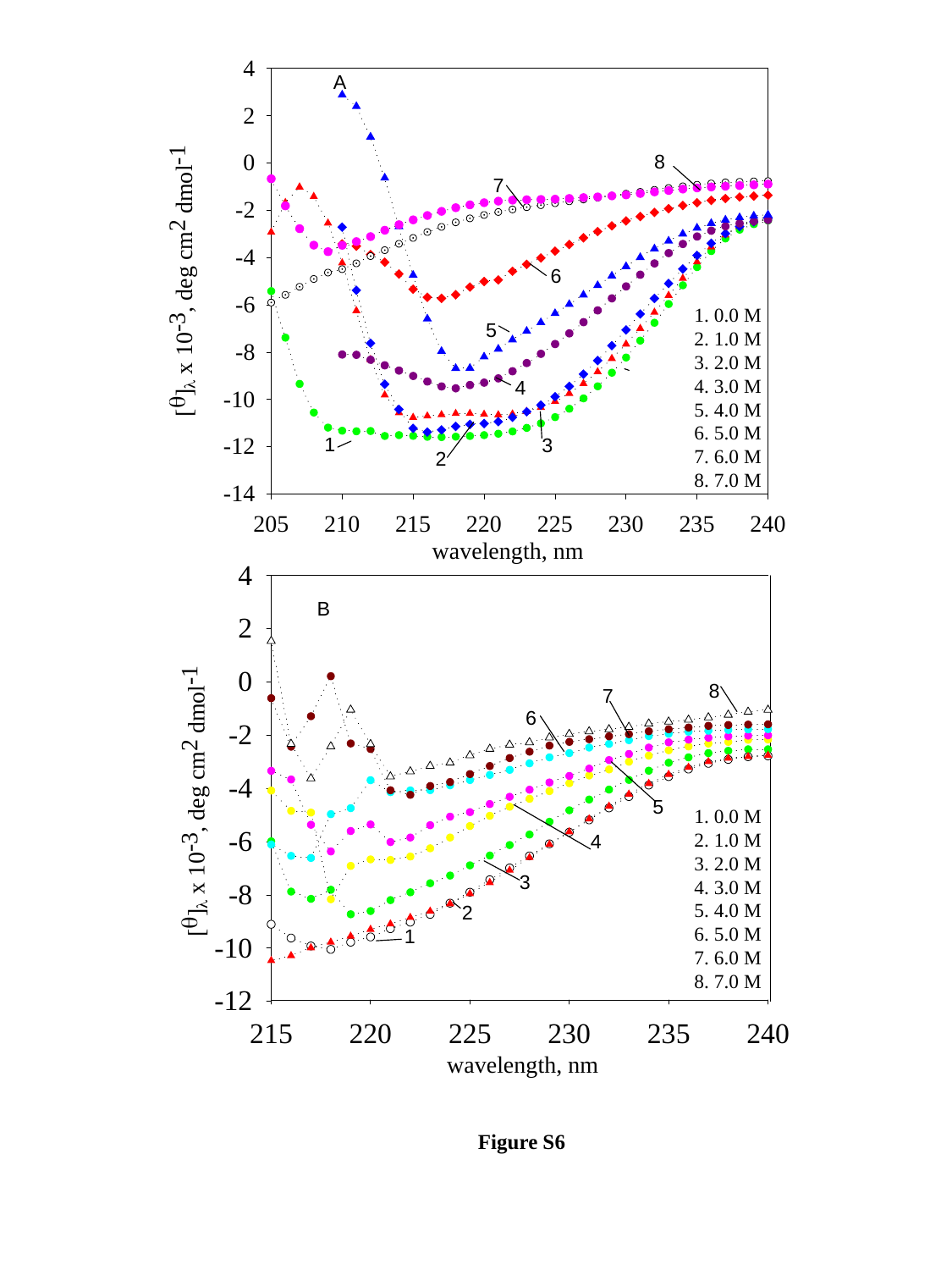

Figure S6

Supplement: S6 Fig — The representative far-UV CD spectra of apo-La at 25°C (A) and at 60°C (B). (PPTX) [file pone.0128740.s006.pptx]

## Slide 1
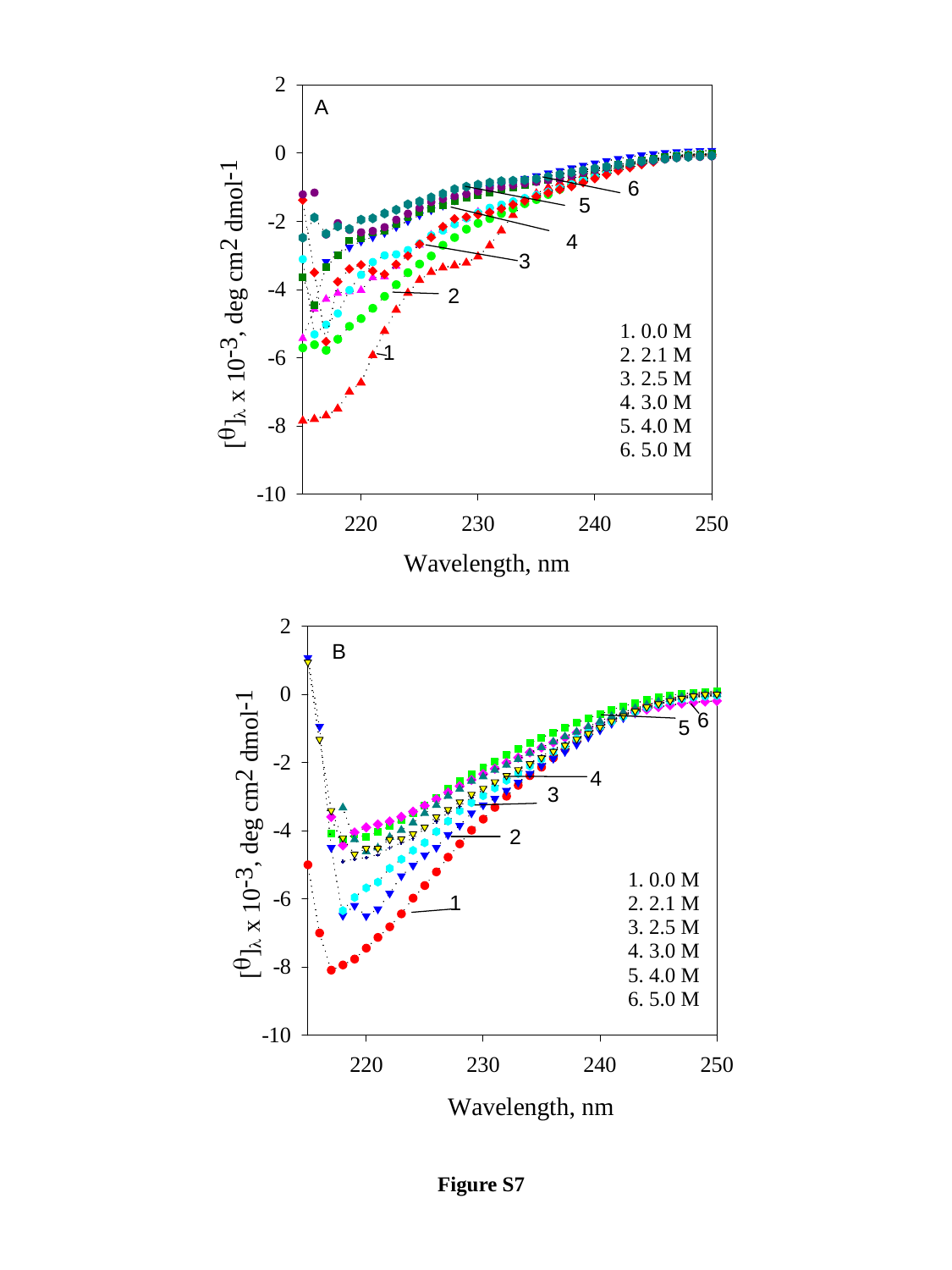

Figure S7

Supplement: S7 Fig — The representative far-UV CD spectra of ctg at 25°C (A) and 60°C (B). (PPTX) [file pone.0128740.s007.pptx]

## Slide 1
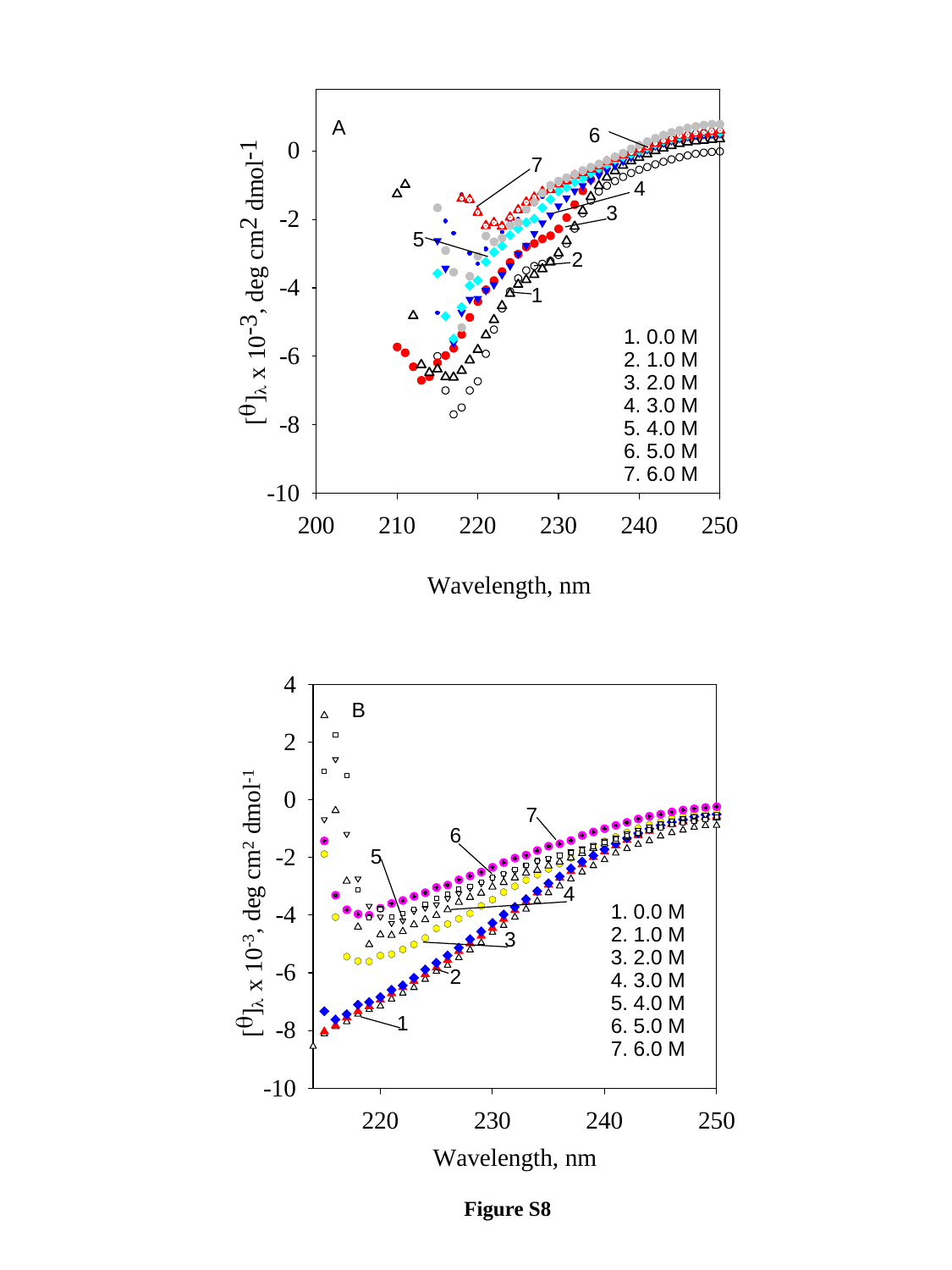

Figure S8

Supplement: S8 Fig — The representative far-UV CD spectra of ctg at 25°C (A) and 60°C (B). (PPTX) [file pone.0128740.s008.pptx]
